# Record of Meteorological Conditions at Buffalo, for July, 1855

**Published:** 1855-09

**Authors:** Sanford B. Hunt


					﻿ART. IX. — Record of Meteorological Conditions at Buffalo, for July,
1855. By Sanford B. Hunt, M. D.
'■	a: i &
P	® A*
c?
PS	Ph	*Q, •	pt
<	a	a ci	g
*	s	S o	”
A A j " x
M	£rg
__________________________________________Sa _________________________
•ajnjBjadcudj, i	g
jo eStiBj jsajBaic)|pi xd	co o x x t— as	d	co	t- °	—	pi	co	pi	ox	to — pi -p to ,o to x	in	in o co
•aji IPlcodt— indent— —	x	—	dtor—pico^in^t—f — c—co — —ci	—	cccopi	m
nrrrmTT ttmtit t?	in	IO C! c; t:	~	n	02 i.C	C.	>	C	C	X-I	c: 7! ('<- X C C. Tj<	—	t- — d	—
~l "llllH iii.o[\ i- x	t~ <-<-t~x	i- z	i-	x	x	o. x	x x x ~c c-. x co	~.	x x t-	x
x	I .copit-; — mcocopimd-ojcidt^comm'^copicoxddmt- to oo t—
S -Mag uvaj\ oocid’i't'OiotOrtrttoNiridcirH.Hcipit'inapiodHcidrHodco in
q __________'toto to to: m to m m to to tototococot—t—t—t—mmm to co e- t— <- t— t- to to to
•ainjB lco.co.coto.mco..cocot-coto to. m	co	co co to co
-Jddaroj, uu.>k x x pi ~r cd ad td ad o ••* cd m o od oo oo’ ci to' ci ci — ■q>' to’ — -4 m x co to m —’ ci
____________t'-	H C C H b- H H H ‘hO H H H
tQ	Cl CO Qi O -—I	OGW'’3’TTr-tO’<T<C'Mc»C-GOOOTl'C'iOOO|0O
•	'A'liDitnnr i	S? i9	T1	2 '“'00	cicocit-otococot-	—	cox —' -P -* -c — t- co —,	— n m	to
s.	z x	>	m ■£> t—	r-xxxdxxxxdxxxddddxxodxxx
5	_________________________________________________________________________________________ l_
§	-lUIOJ AISCT	®	Pl	co t~:	t-;	. CO CO in	.	O CO t— -p Tf — to to .	d d	—
E • a u	d —■	—	x d co	— m -p td — d d d cd	—	d in cd cd -p t^ pi ci d ci	—5 td td	m
; tc	._____to to	to	m m m	to co to to to to t— t— to	t—	t— m up to to to t- co t— co	t- co co	to
_•	~	-any dtnajJ...............................................................................ci
—	lE't2cP ci top? arc - - it io it it ct-1 it c: M i.n toe loin a! ? co « ci
_________c > r- c o c^» o co i> b- h b«	co c h h b- b- h o
g-8^	.	£:	p:	pc £ s M
=	02	32	32 32	02 CQ CO 02 <Z2 S aj 02’020202 S
_________— — — c - o c -i 0 4 e - 4 _ ?j	e e -j o _•
g spnoojoj.tny |o pi o oooooooooooooiooomodot-cocooiajoo ’
S	S £* 2 5° X? & p? 2 <- m ° c- — m t—- to co x t-01 m m — o m^-H- -p
?	•Xiroiuintr f5;’5 ?■!; 71?*	~ ~	c >t a xci P it o o -t ci - - ” t- co
C 14	A""- nH,C- '' C l' O l- (C to I-t' X l'I' to O l' O t'X X Z X O. XX X Z Ct X t-to t—
p	&	-	--------------------------------------------------------
E	‘jntoj Mag	«? a? X m d . . . X) a;	ct Lt p a; rt to x ct i-- > 0. t~ . -p pf co	-P	Pl	.	I co
?	’	S2a^i2a2£2tPt7CQcd—■	ci t^ ci pi cd pi -p cd cd cd zd -p ci cd cd cd	cd cd	pi	x	t—
•	rc	_____to to to to m to mm cocot—	tococot—t—r—t—t—mmmcocor— t— t—	t—t—t—to	to
<=q d?
. — -amr dinar • ..............................................................................co
o ** (' t' ci -r ci ci •« at h z m to to ct i.n rt ct ?i cm z Lt ci o. ct o o. ci -n to
1—1----- _	J- t-t-t- t- t- t- l- I- t- t- OO X 00 00 x to to to to t—r- <- X t- t-_X> X t-
°* g-oSL'g	M >»»»'
tgS§cS af 32’	«2 «2 CQ GO 32 <Z2 02	32 32 32 OC 32’32	(Zj pq 02 02 02 02 02’ 02 02
>	‘“‘Ur-	.................................................
__________O Tj<	CO CO Ct CP CP O? CO CO	O! Pi co cd C5 P> O —'	ci CO P> CO Pl Pl Pl
8Pn°[3Jo?tuiV ao o ci ocico coo as oxpa —>0000 coco o 00	com	moo	co
-pooicooo-p'cicicoi'oco-fpi m x m o c - m o	x x	cTdi -r	ci	x ix
. •jCiTmtnnH ’■£ *-? '* 00 x —i to w to ci pi co <- to x o -p m -r to m — 1-	—" m	co to	co	to m
u +.1. t>toxtor-toxt't't--Ti'XL'XxtfXxa>xx xoxocixcixxxx
® _____________________________________	1
•	§ ‘3UT0A Man •	• .XPl'-|C5COC0t^m-ti< Tf X •# to ■#	XCOCO'rjJtOPI .to-pipix
g	£ ■* • d 11	m — co cd ci m td td -4 -t? to td	o td o’ cd -4 cd o'	odpi-oitf—<—<ouot-4cdcd
6	______totomtommmmmmcototptocot~t~-r-t^co mtototpt—t~cot—toco to
~ 9Jni,dui3T.............................. ......................
-	J- c to x - o o p lp m x - t- - m p z oct ct it	ci cor- — mpix o
f __________i— co to t— to to to co co t— t— t— 1— cot— t—t— <— r— to co co to co t— t— t— r— t— co t—
g-a®. -d	.1
§ § g = r «2 02’02'02 02’GO S 02 02 02 32 S 32	02 CQ 02 02	«2 02 X 32 02 02 f?
O	........................................................
__________Ql~f<*-<OO^G9COOCQCOQ1Q3C3 — CQ Q> C? QI Q? Q? QI Q? — — QJ Qf Q? Q> -h ' _
Bpnoi3jO4.aiv oooooooooooo^ioo^moooooooooocmooio* |
I
•	•Timmer to-ht! . rH Pl Pl Pl COCO — — — Cl — PlCO'djCO — PlCOCPCOCOCOPl— . — Pl Pl —
luo-iuaju i jjQeo^QQQQ^Qcdddodoodoocdddddddd o
Icocpcocococococncocococopicocococococococococncococococpco — co co
«■	o	up	o	co — o — — c- co	to t-	i-
O	_ <-	t-	Cl	Cl— OCICOt— to	d Pl	Cl
■a™H jo -ui	m ■d’	cp-c-pot-rt'oci- m
________ ’_______________________ '	.............. '	-4
.arozri — pi co-cm co t— xdo — pi co-c<mtot-xdo — Ptcc-fintoi-Xdc —
^"<11_____________________________________________________________— — — — — — — — — — PIPIPICIPICIPIPIPIPICOCO
* Fog.
				

